# Study Protocol of MINI SALTEN: a technology-based multi-component intervention in the school environment targeting healthy habits of first grade children and their parents

**DOI:** 10.1186/s12889-017-4327-3

**Published:** 2017-05-06

**Authors:** Irina Kovalskys, Cecile Rausch Herscovici, Paula Indart Rougier, María José De Gregorio, Luciana Zonis, Liliana Orellana

**Affiliations:** 1 International Life Sciences Institute (ILSI), Autonomous City of Buenos Aires, Argentina; 2 Favaloro University, Autonomous City of Buenos Aires, Argentina; 3 University of Buenos Aires, Autonomous City of Buenos Aires, Argentina; 4 Faculty of Health, Deakin University, Victoria, Australia

**Keywords:** Physical activity, Healthy eating habits, Young children, Technology-based intervention, School-based intervention, Obesity prevention

## Abstract

**Background:**

MINI SALTEN is a program developed to increase moderate to vigorous physical activity (PA) and improve eating habits at home and school in first grade children. It aims to assess the effects of a technology family-based and PA school-based intervention. The purpose of this manuscript is to describe the protocol design and the MINISALTEN intervention.

**Methods:**

This is cluster-randomized controlled trial designed to run from July 2015 to November 2016 in 12 public schools of the city of Buenos Aires, matched for socio-demographic characteristics. The intervention is based on two main components: (a) “active breaks” (AB): implemented during school breaks by a PA instructor; (b) “virtual” (V): web-based contents delivered to the families via a multiplatform application. Using a computer generated random sequence participants are allocated to one of four intervention conditions: (AB), (V), (AB + V), and control (C). Outcomes are measured at baseline and 12 months post intervention, and will include data collected from the child and her/his mother/father or guardian. Primary outcome measures are: PA and sedentary behaviour (measured with accelerometers). Secondary outcome measures related are: percentage of kilocalories (kcal) from added sugars, and from total and saturated fats; grams of fruits and vegetables; and number of snacks and kcal coming from their added sugars and total and saturated fats. Family socio-economic level, home environment, and school environment will also be assessed. Statistical analysis is on an intention-to-treat principle. Baseline characteristics are described using summary measures and mixed models (with school as random effect). The effect of the two interventions will be estimated using a generalized mixed linear model with link and distribution selected according to the type of outcome. Included random effects are: child (or mother/father or guardian) accounting for repeated measures; school accounting for cluster induced by school. The most parsimonious model for each outcome will be reported. The False Discovery Rate criterion will be used to correct for multiple testing in non-planned analyses.

**Discussion:**

It is a pioneer assessment of the impact of a technology-based virtual intervention and a school-based PA program, designed to prevent obesity, and involving the parents at public schools of Buenos Aires.

**Trial registration:**

Current Controlled Trials ISRCTN58093412. Registered March 14th, 2016 (retrospectively registered).

## Background

International studies have demonstrated the effectiveness of interventions aimed at improving lifestyle behaviours with the goal of preventing body weight gain and/or improving healthy habits, hence contributing to avoid non-communicable chronic diseases. Nevertheless, most studies represented in the current literature come from developed countries and have been carried out in school settings [1]. In Argentina there is a lack of evidence regarding the effectiveness of physical activity (PA) and healthy eating (HE) habit interventions targeting young school children and their parents in randomized controlled trials using both objective and subjective (questionnaires) methods.

Physical activity during young childhood has been favourably associated to health outcomes regarding cardiovascular risk factors [2–4], body composition [5–7], motor skill development [8, 9], and psychosocial characteristics [10]. Current guidelines recommend that preschool children practice no less than 15 min per hour of at least light intensity physical activity while they are in childcare [11]. By age 6, the recommendation is 60 min each day. For Argentine children, commencement of first grade represents a drastic shift from a kindergarten environment devoid of tables and chairs, in which they freely wander around, to a classroom where they are expected to remain seated at their desk. A complete school day typically goes from 8:30 am to 4:00 p.m.; PA class is bi-weekly (60 min each), breaks are every 45 min (5–10 min each), and playground space is very variable. The level of PA practiced during school hours has been scarcely researched. Moreover, eating habits in school children and their impact on health has been also under-researched, especially in Latin American countries undergoing a process of nutritional transition [12–14].

A direct antecedent to the current study is an intervention that proved successful at improving core dietary behaviours and attitudes, in 10**–**12 year old children attending public schools of the province of Buenos Aires [15, 16]. The alarming obesity prevalence found in this population (21.6%) and previous data on Argentine children [17, 18], indicates that prevention is probably furthered if it commences at an earlier age and involves the parents to promote long-term sustainment of the proposed goals. Argentina is not exempt from the ostensible increase in recent years in the use of smartphones and tablets and evidence indicates that communication based on these new technologies has a strong potential to improve healthcare and clinical interventions in the community [19–21]. However, few of these tools have been validated and there is scarce evidence of their effectiveness regarding health outcomes [22]. Even fewer studies have explored the success of such applications at improving eating habits and at increasing PA [23, 24].

MINI SALTEN (More Jumping and Better Eating at Home and School) is a technology and school-based multicomponent intervention developed to increase moderate to vigorous PA at school and at home and also improve eating habits in first grade children. Evidence from international settings indicates that targeting parental involvement in addition to nutrition and physical activity in the school setting is an important determinant for the success of school-based interventions [25]. The purpose of this manuscript is to describe the protocol design and the MINI SALTEN intervention. The main aim of MINI SALTEN is to assess the effects of a technology family-based and PA school-based intervention on (1) physical activity, and (2) quality of diet and eating behaviour in first grade children of the city of Buenos Aires. The specific aims are to: (a) evaluate the effects of the intervention on children’s specific eating habits (fruit and vegetable consumption, energy intake, snack and breakfast quality), (b) evaluate progression of body mass index (BMI) over time, and (c) identify parental factors associated with changes in PA and in children’s quality of diet. The study hypothesis is that first grade children of public schools of Buenos Aires are more likely to develop healthy habits related to preventing obesity if they are stimulated to active play during breaks and when their parents and themselves are regularly exposed to educational stimuli, delivered virtually.

## Methods/Design

In April 2015, the Ministry of Education agreed to grant access to public schools of the city of Buenos Aires. The Institutional Review Board of the Argentine Medical Association provided ethical approval on 02/25/2015. Informed parent consent/child assent, congruent with local legislation were sought.

### Study design

This is a cluster-randomized trial with four arms defined by a factorial structure of the two components of the intervention: the school-based (active breaks) (yes/no); family-based (web-based or virtual) (yes/no). The four arms of the study are: Active Breaks (AB) intervention, Virtual intervention (V), Active Breaks and Virtual intervention (AB + V) and neither AB nor V, control (C). The unit of randomisation is the school (rather than individual children/families). The study will run during two school semesters along two calendar years (the second semester of first grade and first semester of second grade). Because the enrolment process of families in the study lasted approximately 3 months, the study ran from July 2015 to November 2016.

### Study population and randomization

The Ministry of Education of the city of Buenos Aires pre-selected a pool of public schools, which were invited to participate. Written invitations and an information pack were sent to each school Principal, followed up with visits to recruit schools. Consent was sought from Principals and first grade teachers. The 12 participating schools are located in middle-to low-income areas of the city. The schools were randomly allocated to one of the four intervention conditions using a computer generated random sequence. Blinding was not possible due to the characteristics of the intervention.

While part of the intervention is school-based, the primary outcomes are measured at subject level. Subjects of the study are first grade children. Mothers are also being assessed to evaluate parental factors; the dyad comprised the child and either parent or guardian in case the mother can’t attend the interview. All parents of first grade students (typically aged 6) of participating schools were invited to attend a meeting in which the nutritionist presented the program and described the study as well as the type of participation expected from parents and children. Those expressing interest were invited to enrol in the study and an initial face-to-face interview to be held at school was scheduled. Dyads were excluded if the child had severe intellectual difficulties or limitations to engage in physical activity, suffered from illnesses compromising nutrition or food selection, was taking medication known to affect body weight; or if the participating adult had severe intellectual difficulties. After screening for eligibility, the interviewer briefly explained the purpose and characteristics of the program and those who agreed to participate signed the informed consent form. Because the study is web-based, families without Internet access (at home, work or in the neighbourhood) are discouraged from participating. Should they still express wish to participate their data is collected but will be excluded from the analysis. Dyads attending a given school were assigned to the intervention condition randomly allocated to that school.

### The intervention

The intervention is framed within the ANGELO (Analysis Grid for Elements Linked to Obesity) ecological model [26], which promotes environmental modifications encompassing the physical, economic, political, and/or socio-cultural realms, especially with regard to what is available, what are the costs, what are the rules, and what are the attitudes and beliefs at play in the adoption of healthier eating and PA habits. When designing the web-based presentations and activities, low cost activities and argentine family customs are taken into account as well as the attitudes and beliefs targeted to encourage behavioural changes (HE and PA) of children and their parents, especially their mothers. Implementation of the protocol is relied on two intertwined pillars: 1-accessibility/ availability (of practicing PA by promoting active breaks) and 2-knowledge/education (delivered by the virtual intervention).

The MINI SALTEN interventions are based on two major components:

1. Virtual (V). Especially designed web-based contents, targeting families and children aimed at 1) encouraging the improvement of eating habits of the whole family both at home and at school, and 2) increasing mothers’ knowledge of the benefits of PA and consequences of sedentary behaviour, and proposing pleasurable movement entailing activities for the children. Thematic goals are: breakfast, PA, water intake, fruit and vegetable consumption, energy balance, parents as models, and snacking. The contents are produced, developed and structured by nutritionists and specialists in education. The technical team of Nearpod, provides advice on the design and technical support for uploading the theoretical units.
  The material mainly comprised information on the benefits entailed in attaining the proposed goal, tips on how to overcome the particular environmental barriers, and recommendations regarding its implementation (Table 1). This material is delivered through a multiplatform application (https://nearpod.com/) consisting essentially of a personalized virtual campus in which videos and PowerPoint presentations can be shared with the viewer and interactive activities designed to motivate their compliance were proposed. The platform was originally developed as an interactive educational tool, for teachers within the classroom. For the purpose of this research, adaptations are made so that families can receive the presentations on their smartphone, tablet, or computer, and view them during their free time, ideally at home. .
  Each thematic goal is addressed in 2–5 sequential presentations. Families are expected to interact with 23 presentations during the length of the program. It is important to note that although the web contents target mothers, the rest of the family (parents and siblings) are able (and invited) to access the virtual content.
2. Active Breaks (AB). Activities comprising movement during school breaks are promoted with motivation as a backbone and devoid of a competitive strive; active play in which fun, pleasure, and sharing were emphasized. PA instructor-guided activities take place three times a week during two school breaks with the goal of promoting active play for a minimum of 20–30 min/day, depending on each school. The intervention staff provides, simple, basic, affordable elements to be implemented always during school breaks and in PA class (the latter only if the teacher is willing). All first grade children of the enrolled schools allocated to receive the AB intervention participate in this activity. MINISALTEN developed a blueprint of suggested 15 playful games and activities designed to encourage moderate to vigorous PA for children ages 5–7. The goal is that children spend 75% of their time running or jumping (e.g. running in zigzags while chasing a “mouse”; climbing and crouching imitating animal movements, rope jumping, flying like a fish, etc.). The 6 PA instructors rotate among schools to ensure that any individual facilitator aspect is incorporated to all participating schools. Specific gear, likely to increase the child’s level of PA is provided to this effect. This school-based intervention is complemented with a partial version of the web-based program. Its content dealt with the benefits of being physically active, tips on how to reduce screen time, statistics regarding sedentary behaviour, a description of PA recommended for a child, frequently encountered environmental barriers, and how to overcome them.

**Table 1:** Themes and theoretical content of material delivered via the multi-platform application

Theme	Number	Theoretical content
Program presentation	1	Description and purpose/value of MINI SALTEN.
Breakfast	4	Benefits, variety and quantity of food items, barriers, and tips for implementing.
Physical activity	4	Importance of being physically active, recommendations, barriers, and tips for implementation.
Parents as role models	2	Concept illustrated with examples.
Water intake	2	Importance of hydration for the body and how to recognize dehydration.
Energy balance	2	What it is and why it needs to be balanced.
What we eat	1	Food groups, amounts, and examples of healthy menus.
Fruit and vegetables	5	Benefits, tips, recipes, importance of colours, seasonal fruits and vegetables.
Snacks	2	Suggested healthy and affordable snacks for school. Frequency and variety.

*N* number of interactive presentations of each theme

Both energy expenditure and energy intake are crucial components of energy balance [27]. Active breaks at school are a window of opportunity to increase energy expenditure. The virtual web- based program is aimed at instructing the child and family on nutrition with the ultimate goal of improving the other component of the energy equation.

Process evaluation is supervised by a coordinator who monitor attendance and fidelity of implementation of the intervention component delivered by the PA instructors, as well as compliance with the expectation that activities practice during breaks be of moderate to vigorous intensity during 80% of the allocated time. This is ensured through careful planning and daily registry.

The amount of time assigned to this intervention results from the number of school days that are planned for intervention minus the days children do not attend for different reasons (holidays, elections, premises’ disinfection, etc.), or those in which the PA instructors are unable to deliver the intervention for any other reason.

All participant first grade children of the 12 enrolled schools will be assigned to one of the interventions: Active Breaks (AB), Virtual (V), Active Breaks and Virtual (AB + V) and control (C) group. Based on the aforementioned components four types of intervention were designed.

#### Control schools

College students of nutrition will deliver two educational workshops for parents once the intervention and assessment is completed (one HE and one on PA). These workshops will be crafted with the additional goal of showing appreciation to the parents for their participation in the assessment (thus enhancing the study’s retention rate), and to improve the school staffs’ interest and commitment in its support of the periodic assessment.

### Data collection

Due to logistic reasons the participating schools will be join the program in a series of steps. The first assessment interview (baseline) will take place sometime between June and October 2015. The second assessment interview (at the end of the intervention) will take place 12 months later. In the few cases where the adult participant of the dyad is different from the mother (typically a father), he will not be included in the anthropometric assessment.

Data will be be collected via questionnaires and objective measurements. The child is only subject to the latter; only the mother will respond to the former. The questionnaires will be administered in face-to-face interviews with the participating mother. After ending the baseline interview, mothers will receive instructions regarding the use of the accelerometer with a diary to be filled out by the mother during the 7 consecutive days of use. Eight days later, a research assistant will retrieve the child’s accelerometer at school.

### Measures

Detailed and specific measures for each dyad member and assessment time are shown in Table 2.

**Table 2:** Assessment methods, timeline, and outcomes

Measurement	Source	Measure or instrument	Outcomes
Anthropometry	Child	Weight (kg), height (cm), waist circumference (cm)	BMI Z-score, overweight, obesity and central obesity.
	Mother (or father or guardian)	Weight (kg), height (cm), waist circumference (cm)	
Blood Pressure	Child	Blood pressure (mm Hg)	Classification of blood pressure (high, normal or low).
	Mother (or father or guardian)	Blood pressure (mm Hg)	
Physical Activity (PA) and Sedentary Behaviour	Child	Accelerometer	Accelerometer: Daily steps, minutes of moderate to vigorous PA (min/day), weekly METs (week days and week-end days) and sedentary time.
	Mother (or father or guardian)	Global Physical Activity Questionnaire (GPAQ - WHO)	GPAQ: sedentary behaviour and PA at work, during leisure, and for transport (min/day).
Dietary Intake	Child	24-h Recall using the Multiple Pass Method (completed by parent/guardian)	Daily caloric intake (Kcal/day), breakfast quality (mg calcium/day), portions of fruits and vegetables, volume of water (ml/day), and percentage of calories from saturated fats and of grams of added sugar coming from snacks.
	Mother (or father or guardian)	24-h Recall using the Multiple Pass Method	
Socio-Economic Status	Mother (or father or guardian)	Modified and adapted version of SAIMO Socio-Economic Level Survey	Classification of social and economic status (low, middle or high).
Home and Neighbourhood Environment	Mother (or father or guardian)	Modified and adapted version of ISCOLE Neighbourhood & Home Environment Questionnaire	Neighbourhood social capital, home social environment, home and neighbourhood food environments, home and neighbourhood PA environment and neighbourhood facilities environment.
School Environment	School Principal (or Vice- Principal, or School Secretary)	Modified and adapted version of ISCOLE School Environment Questionnaire	School facilities, healthy eating and PA policies, extracurricular activities, frequency of physical education and breaks, promotion of active transportation and the availability of healthy and unhealthy foods.

#### Physical activity

The child’s PA and sedentary behaviour will be objectively assessed at baseline and 12 months with a tri-axial accelerometer (model GT3X+ ActiGraph, Pensacola, FL, USA). Accelerometer data will be collected and then downloaded in 15-s epochs. The accelerometer will be worn 24 h per day for 7 consecutive days (including the weekend days), placed on an elasticised belt at the waist on the right mid-axillary line. Mothers will be instructed on the placement and use of the accelerometer so as to guarantee correct compliance with the procedure. This will be done during the face to face interviews conducted at baseline and 12 months. They will be also instructed to remove the device when the child practiced water activities (bathe, swim or water sports). To guarantee correct compliance with the procedure in addition to receiving printed instructions, they will be contacted by the research team while the child is wearing the device in order to ensure proper use and to discuss any issue or doubt regarding information recording. The minimal acceptable amount of accelerometer data is 5 days including at least 1 weekend day; with at least10 hours of wear time per day. Periods of 60 or more minutes of continuous zeros will be considered no-use-times and will be excluded from analysis. After retrieval of the device, the research team will verify the data for completeness using the ActiLife 6.11.8 software (ActiGraph, Pensacola, FL, USA).

Accelerometer outcomes are: daily steps, minutes of PA, intensity of moderate to vigorous PA determined by Evenson et al.’s cut points and weekly METs during week days and during week-end days [28]. Evenson’s cut points define sedentary time that ranges from 0 to 100 counts per minute (cpm). In children of 5 to 8 years using ActiGraph accelerometers, the following classification is proposed: sedentary (0–100 cpm), light (101–2295 cpm), moderate (2296–4011 cpm), and vigorous (≥ 4012 cpm). These counts are based on 60-s epochs**.** Sedentary time and sleep duration will also be analysed. The ActiGraph accelerometers have the “inclinometer” function that determines the child’s position: sitting, standing, and lying down [29–31]. Sleep time is defined as the period elapsed from nocturnal sleep onset to nocturnal sleep offset, including all minutes scored as sleep or wake. Sleep period time for each child will be determined using a fully-automated algorithm specifically developed for use in the ISCOLE study and other epidemiological studies employing a 24-h waist-worn [29].

The mother’s PA and sedentary behaviour will be assessed with the Global Physical Activity Questionnaire (GPAQ), endorsed by WHO for surveillance of PA at a community level especially for developing countries (http://www.who.int/chp/steps/GPAQ/es/). It has acceptable validity and reliability; is able to adapt to take into account country’s cultural differences, and assesses information on sedentary behaviour and on PA at work, during leisure, and for transport [32, 33].

#### Dietary intake

Child’s and participant parent dietary intake and habits will be assessed at baseline and 12 months with a 24 h recalls using the Multiple Pass Method. This interview will be carried out at school, in a private space provided by the institution. A nutritionist will conduct the 60-min face to face interview with participating parent collecting the parent’s dietary intake and then the child’s [34]. A Visual Guide of Food’s Weight and Portions size will be used to help the parent identify the amount, portion size, weight, volume, size of the food item, and amount of liquid consumed the prior day [35]. ILSI Argentina contributed to the development of this support material and all the information obtained with this instrument will be analysed with the Nutrition Data System for Research software, version 2013 (NDS-R, Minnesota University, MN, USA) NDS-R software. Daily dietary intake outcomes are: daily caloric intake (Kcal/day), breakfast intake (yes/no), portions of fruits and vegetables, volume of water (units), and percentage of calories from total and saturated fats and of grams of added sugar coming from snacks.

### Anthropometric measures

#### Child and mother

Anthropometric measures will be taken at baseline and 12 months according to standardized procedures. Weight will be measured to the nearest 0.1 kg using a portable digital scale (SECA AURA 807) wearing light clothes. Height will be measured to the nearest 0.1 cm and with a portable stadiometer (SECA 206. Hamburg, Germany), with participant fully erect, without shoes, feet together, head in the Frankfort Plane and at the end of a deep inhalation. Waist circumference will be measured to the nearest 0.1 cm at the end of gentle expiration with a non-elastic anthropometric tape held midway between the lower rib margin and the iliac crest (Sanny, Berasil), utilizing the hands crossed technique in mid-waist for adults (WHO) and in minimal waist for children [36]. Central obesity in adults will be defined for each sex according to WHO recommendations [37] (females: > 88 cm; males: >102 cm). In children, central obesity will be defined using Taylor et al.’s cut-offs. [36]. The BMI Z-score will be calculated using the WHO reference growth charts for children [38] and for adults.

Each measure will be repeated, and the average will be used for analysis.

Blood pressure will be assessed at baseline and 12 months using a digital sphygmomanometer OMRON HEM-7114EF), with the appropriate cuff (adult or child). [39]. For children, the measures will be classified by age and height percentile. Adults’ measures will be classified according to international [40] and national [41] recommendations.

#### Environmental measures

Neighbourhood & home environmental characteristics will be assessed once, at 12 months post intervention with a modified and adapted version of the ISCOLE Neighbourhood & Home Environment Questionnaire administered to the mother [42]. It informs about the support given by the neighbours and family members as well as of the food and PA environment both of the home and neighbourhood. Social and economic status will be assessed with an adapted version of the SES Survey [43]. School environment will be assessed with a modified and adapted version of the ISCOLE School Environment Questionnaire [42]. This instrument will be administered to the headmaster, and assesses the school’s facilities, as well as the schools’ rules and current practices with regard to HE and PA.

One week after first evaluation, each participant parent will be e-mailed the first segment of the targeted content uploaded to the aforementioned multi-platform. The application was designed to register every visit so that the progress/hurdles regarding implementation of the proposed goals could be monitored on a one-to one basis. In order to enhance motivation and assess compliance, the content also inquired about the family’s habits and the children were able to interact with the platform via drawings and games (e.g. Memotest, etc). It is possible to view parental responses to the interactive questions, as well as the child’s drawings. If the parent failed to log in to the application within due time, she/he will be contacted via text message, or e-mail in order to be assisted in staying on track. The subsequent presentation will be forwarded 2 weeks after the presentation has been visited.

The efficacy of the intervention will be evaluated through outcomes measured on the children. Primary outcomes: (Aim 1 -PA): 1) Total time (minutes/day) of children’s moderate to vigorous PA and 2) Sedentary time (minutes/day). Secondary outcomes: (Aim 2 - Quality of diet): 1) Percentage of kilocalories (kcal) from added sugars, 2) Percentage of kcal from total fats and saturated fats, 3) Grams of fruits and vegetables, and 4) Number of snacks and kcal from total fats, saturated fats, and sugars coming from snacks. The 4 secondary outcomes will be measured by a 24-h recall, using the multiple pass method, applied to both mother and child and complemented with information regarding school breakfast and lunch in order to collect intake from an ordinary school day, covering both home and school.

### Statistical considerations

**Fig. 1:**
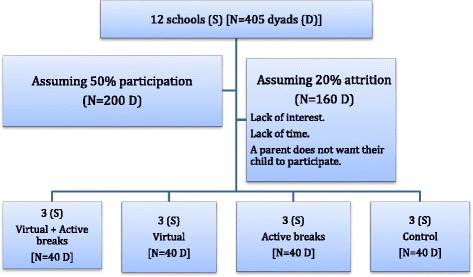
Flow Diagram

Sample size and power calculations. The number of children enrolled in first grade in the 12 participating schools is 405 [(range: 11–55, mean = 35]. Assuming approximately 50% participation rate the aim is to enrol a minimum of 200 dyads, i.e. 50 per trial arm. Assuming an additional 20% attrition it is expected to collect data on 160 dyads (40 per arm, an average of 13 per school) (Fig. 1). Under a factorial ANOVA design with two factors (V and AB interventions), and each at two levels, the target sample size of *N* = 160 participants (40 in each of the four conditions) yields a 80% power to detect an effect size of 0.22 (Cohen’s f low/moderate) for any of the main effects (V, AB) and the interaction effect (V*AB); F test, α = 0.05.

An effect size of 0.22 corresponds to a change of 5 min/day in moderate/vigorous PA (within-cell SD = 11) and a change of 5.4 min/day of sedentary time (within-cell SD = 12) [44].

Statistical analysis will be conducted on an intention-to-treat principle. Therefore, all children will be included in the analysis regardless of the intervention uptake. However, the percentage of “attendance” (compliance) of each child/family can be measured and will be used for subsequent analyses to evaluate the intervention uptake by the families.

Baseline characteristics will be described using summary measures and mixed models (with school as random effect), selected depending on their distribution. The effect of the two interventions on each one of the study outcomes will be estimated using a generalized mixed linear model with link and distribution selected according to the type of outcome. The model will include “active breaks” (AB), “virtual” (V), active breaks and virtual (AB + V), time (baseline/end of study) and the interactions time*AB, time*V and time*AB + V as fixed effect. It will also include random effects: child (or mother) to account for the repeated measures, and school to account for the cluster induced by the school. The most parsimonious model for each outcome will be reported. The False Discovery Rate criterion will be used to correct for multiple testing in non-planned analyses.

## Discussion

To our knowledge, this is the first study to assess the impact of a communication and technology-based virtual intervention involving the parents of first grade children of public schools in Latin America and designed to prevent obesity in their families. If this resource should prove to be effective, and taking into account the widespread use of new communication technologies, it may contribute to the dissemination and promotion of health related habits, and ultimately aid in the fight against the challenge posed by obesity. The additional value of this tool is that it is amenable to be replicated and implemented at a distance, hence resulting cost effective.

Because all measurements will be obtained according to standard methodology and protocols, particularly the objective measures of PA with accelerometers, an added value of data to be reported by this study is that it is one of the few to provide unbiased information regarding dietary and PA patterns of first grade children of Argentina.

The theoretical model that pervades the program emphasizes accessibility and knowledge as critical pillars of change. The first one is addressed by providing an increased number of opportunities for practicing PA during school breaks; the latter in the provision of knowledge about healthy habits related to food intake and PA. Assessment of a theoretical model in which the components are split off and each one of them is evaluated both separately and then together with an objective methodology, will enable us to identify more precisely the value of each of the components that traditionally are included in a multi-component intervention aimed to prevent obesity. Developing countries are lacking information on interventions that are effective in the prevention of non-communicable diseases. Studies of this kind should be able to provide the scientific community and public health entities with tools that are both efficacious and evidence-based.

There is a general agreement about the importance of developing tools that contribute to prevent chronic diseases affecting global health. Regarding eating habits and behaviours linked to physical activity, the scientific community has agreed with the importance of beginning as early as possible, of including families, and if possible, the whole community in preventive strategies. An important barrier to the inclusion of parents in school preventive programs is their lack of time. Current technology appears to be an auspicious and affordable solution to reach them.

## Abbreviations

AB: Active breaks
AB + V: Active breaks and virtual
BMI: Body mass index
C: Control
GPAQ: Global physical activity questionnaire
HE: Healthy eating
PA: Physical activity
V: Virtual
WHO: World Health Organization
